# Characterization of Micro-Holes Drilled Using a UV Femtosecond Laser in Modified Polyimide Flexible Circuit Boards

**DOI:** 10.3390/mi15091078

**Published:** 2024-08-26

**Authors:** Lijuan Zheng, Shuzhan Lin, Huijuan Lu, Bing Huang, Yu Liu, Jun Wang, Xin Wei, Jun Wang, Chengyong Wang

**Affiliations:** 1 Institute of Manufacturing Technology, Guangdong University of Technology, Guangzhou 510006, China; 2 State Key Laboratory for High Performance Tools, Guangzhou 510006, China; 3 Zhuhai Jingwang Flexible Circuit Co., Ltd., Zhuhai 510006, China

**Keywords:** flexible printed circuits, micro-holes, femtosecond laser, processing quality

## Abstract

Modified polyimide (MPI) flexible printed circuits (FPCs) are used as chip carrier boards. The quality of the FPC directly affects the reliability of the integrated circuit. Furthermore, micro-holes are critical components of FPCs. In this study, an ultraviolet (UV) femtosecond laser is used to drill micro-holes in double-layer flexible circuit boards with MPI as the substrate. The morphology of the micro-hole wall in the copper foil and MPI layer is observed, and the effects of the laser processing parameters on the diameter and depth of the micro-holes are analyzed. The drilling process and mechanism of micro-holes obtained using a UV femtosecond laser in MPI FPCs are discussed. The results show that the morphology of femtosecond laser-machined copper is closely related to the laser energy, and a periodic structure is observed during the machining process. Copper, MPI, and copper oxides are the most common molten deposits in micro-holes during drilling. The depth of the micro-holes increases with an increase in the energy of a single pulse, scanning time, and scanning overlap rate of the laser beam. However, the diameter exhibits no discernible alteration. The material removal rate increased significantly when laser processing was applied to the MPI resin layer.

## 1. Introduction

Flexible printed circuits (FPCs) have many benefits, including high wiring density, low weight, small thickness, and folding and bending capabilities [1]. FPCs serve as essential infrastructure for signal transmission in transceivers and as power supplies in consumer electronics, automotive electronics, industrial control, medicine, and other end-use electronic systems [2]. FPCs are fabricated from polyimide (PI) resin or modified polyimide (MPI) resin as an insulating substrate and laminated with copper foil. They are typical metal–polymer heterogeneous composites, exhibiting low overall stiffness and significant variance in longitudinal thermal conductivity and thermal expansion coefficients [3].

After copper plating, the micro-holes of the flexible board enable line connection, with the signal transmission capacity and quality determined by the micro-hole processing quality. Flexible boards can be categorized as single-sided, double-sided, or multi-sided. Flexible boards with micro-holes require the processing of composites made of copper foil and other polymers that are alternately pressed together. The holes must be dense and small, and the surface quality and dimensional deviation must be maintained within 10% of the machining process depth. Therefore, FPC micro-hole processing faces major obstacles [4]. Currently, two primary methods are used for FPC hole processing: laser drilling and mechanical drilling. Through-holes with a diameter of 0.1 mm or more undergo processing using mechanical drilling, whereas micro-holes with a diameter of 0.125 mm or less are processed using laser drilling [5]. Consequently, laser processing is more broadly used for micro-hole processing of flexible boards than mechanical processing methods.

Currently, the two most commonly used lasers for drilling are 930–1064 nm CO_2_ lasers and ultraviolet (UV) lasers with wavelengths in the range of 266–355 nm. CO_2_ laser processing has a significant impact on temperature because the copper foil absorbs a considerable amount of light and must be chemically browned before processing [6]. As an alternative, segmented laser processing, where the subsequent layer of material is processed using a CO_2_ laser, can be utilized using other lasers after Cu has been removed. Metal and polymer material alternation is a challenging and intricate procedure to achieve using CO_2_ laser processing. Instead, UV laser processing can be used to produce micro-holes that are smaller and of higher quality. UV lasers have a wide range of applications in the fields of communications, electronics, and medicine [7]. The two primary components of the UV laser ablation mechanism are photothermal and photochemical reactions [8]. The term “photothermal reaction” describes how the material absorbs more laser energy, melts, and vaporizes, producing a heat-affected zone and a visible recast layer on the aperture and aperture wall. The term “photochemical reaction” describes the process where less heat is deposited on the substance being processed and the chemical bonds of the material are broken when the laser photon energy is higher than the molecular bonding energy of the material.

In recent years, long-pulsed lasers have been frequently utilized to produce flexible panels. However, because their pulse widths are larger than the thermal diffusion time of the material, long-pulsed lasers can result in problems such as microcracks, poor pore shapes, and heat-affected zones. Ultrashort-pulse lasers, which are widely used in composites, metals, and polymer materials, have pulse durations ranging from picoseconds to femtoseconds. In addition, the material restriction is lower. This enables them to minimize thermal damage during micro-hole machining.

Existing research on micro-hole processing for FPCs mainly focuses on single-layer flexible boards, with minimal research on double-layer flexible boards; furthermore, PI resins are mainly used in FPCs rather than MPI. Zhao et al. [1] used picosecond lasers with wavelengths of 355, 532, and 1064 nm to process through-holes with diameters of less than 10 µm and hole spacings of 0–2 µm on a single-layer flexible board. Lu et al. [9] removed the top layer of copper foils without damaging the lower layer of the PI by processing a single-layer flexible board using a UV femtosecond laser. This allowed lines with widths of less than 45 µm to be processed. Few existing studies have focused on FPCs as composite materials and further research can be conducted by examining the characteristics of laser processing of copper and resin to further analyze their processing characteristics. In terms of the surface morphology of laser-processed copper, Biswas et al. [10] used a 275 nm femtosecond laser to process copper and found that the structure of the copper surface changed from periodic ripple to stacks with an increase in the cumulative energy density. Anderson et al. [11] compared infrared femtosecond and picosecond laser pulses in the formation of self-organized surface features on copper and found that femtosecond laser processing resulted in the formation of inhomogeneous structures on copper. In terms of the effect of laser processing parameters on copper, Han et al. [12] conducted a coupling study of various parameters on 280 µm-thick copper and found that the laser power and number of scans had a significant effect on the micro-holes taper and circularity. The hole taper resulted from the Gaussian distribution of the laser energy, laser divergence, and multiple reflections of the laser in the pits. Finally, micro-holes with a diameter of 1 mm, a taper of 2.556°, and a roundness of 0.992 were processed. Balachninait et al. [13] investigated the removal ratio of copper as a function of pulse energy, with varying energy distributions within single and multiple pulses. Multi-pulse ablation resulted in a significant reduction in the removal ratio of copper compared with that of single-pulse ablation of grooves.

Currently, only a few studies have investigated the laser processing of MPI, most of which focused on the laser processing characteristics of PI, processing mechanisms, and effects of processing parameters. Lorenz et al. [14] used a UV picosecond laser to process micro-holes in PI films with a thickness of 13 µm and a diameter of 0.7–75 µm, achieving high precision porosity with a pore size standard deviation of less than 10%. Zhang et al. [15] used a UV nanosecond laser to study the effects of laser power, scanning speed, and their combination on the ablation behavior of PI and found that photothermal and photochemical reactions occurred simultaneously during the laser processing of PIs. During pyrolysis, the main chain decomposed and rearranged itself, and CO_2_ was released at 647 °C. Hu et al. [16] used a UV nanosecond laser to pattern 0.125 mm PI films and found that the laser beam penetrated the PI film and began to irradiate the metal platform underneath the PI film when the laser power density was sufficiently high. The metal platform started absorbing energy, warmed up, and then transferred the energy to the lower surface of the film, which accelerated the extension of the width at the exit, resulting in an exit width larger than the entrance width.

In general, few studies have examined the micro-hole laser processing of double-sided flexible plates formed by the Cu–MPI–Cu composite. In this study, we used a UV femtosecond laser to process the micro-holes of a double-sided flexible board. Furthermore, the material removal procedure of laser-machined flexible boards was analyzed, the morphological features of the three stages of laser removal of the composite material were examined, and the effects of the laser single-pulse energy, number of processing times, and pulse overlap (PO) rate on the diameters and depths of the micro-holes were evaluated. This study provides a theoretical basis for the micro-hole laser machining of FPCs using MPI as the substrate.

## 2. Materials and Methods

### 2.1. Experimental Equipment and Materials

**Figure 1:**
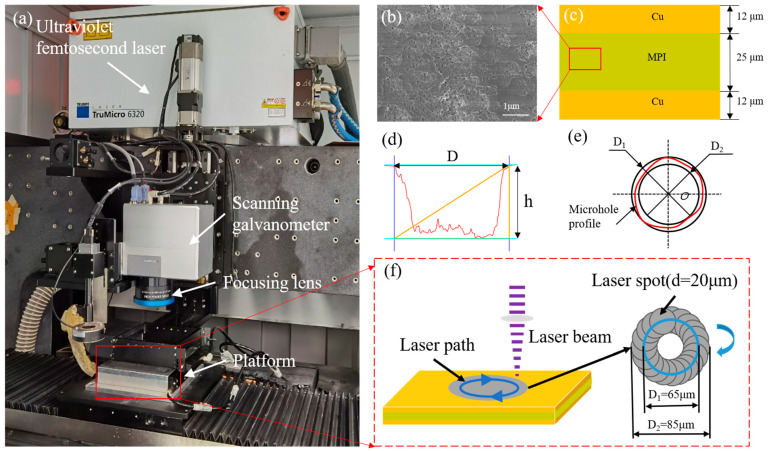
Experimental setup. (**a**) Processing system for the UV femtosecond laser; (**b**) modified polyimide resin substrate electron microscopy; (**c**) diagrammatic representation of the FPC cross section; (**d**) diagrammatic representation of micro-holes depth measurement; (**e**) diagrammatic representation of micro-hole diameter measurement; (**f**) diagrammatic representation of micro-hole drilling.

A PM1609150064 UV femtosecond laser processing system, made by Shengxiong Laser Advanced Equipment Company, Dongguan, China with a wavelength of 343 nm and pulse width of 500 fs, was used in the micro-hole drilling experiment for the femtosecond laser processing of double-layer flexible circuit boards. As shown in Figure 1a, the energy distribution of the laser is Gaussian and its polarization state is linear; the relevant parameters of the experimental equipment are listed in Table 1. The double-layer FPC is provided by Jingwang Flexible Circuit Company, Zhuhai, China. The model is FB12-25-12UEY. The double-layer FPC has a total thickness of 49 µm and was composed of 25 µm MPI resin and 12 µm electrolytic copper foil that were pressed together at the top and bottom, as shown in Figure 1b,c. MPI is produced by modifying conventional PI for processing. For instance, PI is difficult to process, with an extremely high curing temperature and a synthesis process that results in high defects. MPI has a lower dielectric constant and loss factor compared with PI, which helps to increase the propagation speed of signals in the material and improves the efficiency and performance of circuits.

### 2.2. Experimental Design

FPCs were drilled with a femtosecond laser at a single-pulse energy of 3.4 μJ, a PO rate of 98.33%, and 23 processing periods to examine the material removal process. The number of processed laps, PO rate, and single-pulse energy were selected as variables to evaluate the effects of the processing parameters on the depth and diameter of micro-holes, as presented in Table 2. PO is defined as the overlap of two successive laser beam pulses on the surface of the material. The PO rate is calculated as follows:

(1) $$PO=(1-\frac{V_{x}}{fd})100\%$$

where *V_x_* is the processing speed, *f* is the frequency, and *d* is the spot diameter.

The material removal rate (MRR) is defined as the volume of material removed by the laser per unit time and is calculated as follows:

(2) $$MRR=\frac{V}{T}=\frac{\frac{1}{4}h\pi D^{2}}{T}=\frac{h\pi D^{2}}{4T}$$

where *V* is the material removal volume, and the volume of a cylinder is used to approximate the volume of the laser-processed micro-holes. *T* is the total laser-processing time for the micro-holes. *D* is the diameter of the laser processing, and *h* is the depth of the laser.

(3) $$T=\frac{D}{V_{x}}(\frac{D}{\Delta y}+1)\cdot N_{s}$$

where Δ*y* is the offset distance between two adjacent scanning paths in the Y direction, and *Ns* is the number of processing times.

The diameters and depths of micro-holes are calculated as shown in Figure 1d,e. The maximum diameter D1 and minimum diameter D2 must be measured to determine the micro-hole diameter. The micro-hole machining diameter is determined by averaging several measurements. The machining diameter of the micro-holes is 85 μm. The laser removes the material in a circular motion, and, as the number of passes increases, the intermediate material is dislodged to form the through-hole, as shown in Figure 1f. Since the diameter of the laser spot is 20 μm, the diameter of the laser circular motion of the processing method is 65 μm.

### 2.3. Experimental Testing Methods

A Hitachi high-tech ultra-high-resolution field emission scanning electron microscope (SEM) SU8200 Series (Hitachi High-Tech, Tokyo, Japan) was used for microscopic observation of the micro-hole morphology and energy spectrum composition analysis. Because of the excellent insulating qualities of the MPI resin material, the flexible board needs to be sprayed with gold before examination using an SEM to view a clean image. To prepare the micro-hole section of the sample, we mixed the metallographic adhesive powder with the curing agent in a 1.5:1 proportion to form a curing solution. Subsequently, the sample and the curing liquid were poured into a mold, which was demolded when the liquid in it had solidified. The sample was then ground using a metallographic grinder until the micro-hole cross-section reached the required smoothness and clarity. The diameter and depth of the sample surface of the laser-processed micro-holes were measured using a noncontact approach, namely, using the Olympus laser confocal microscope (Olympus Corporation, Tokyo, Japan).

## 3. Results and Discussion

### 3.1. Morphology Analysis of Micro-Hole Formation in Phase Field Crystal Processed Using a Femtosecond Laser

#### 3.1.1. Analysis of the Micro-Hole Surface Morphology Characteristics of FPCs Processed Using a Femtosecond Laser

**Figure 2:**
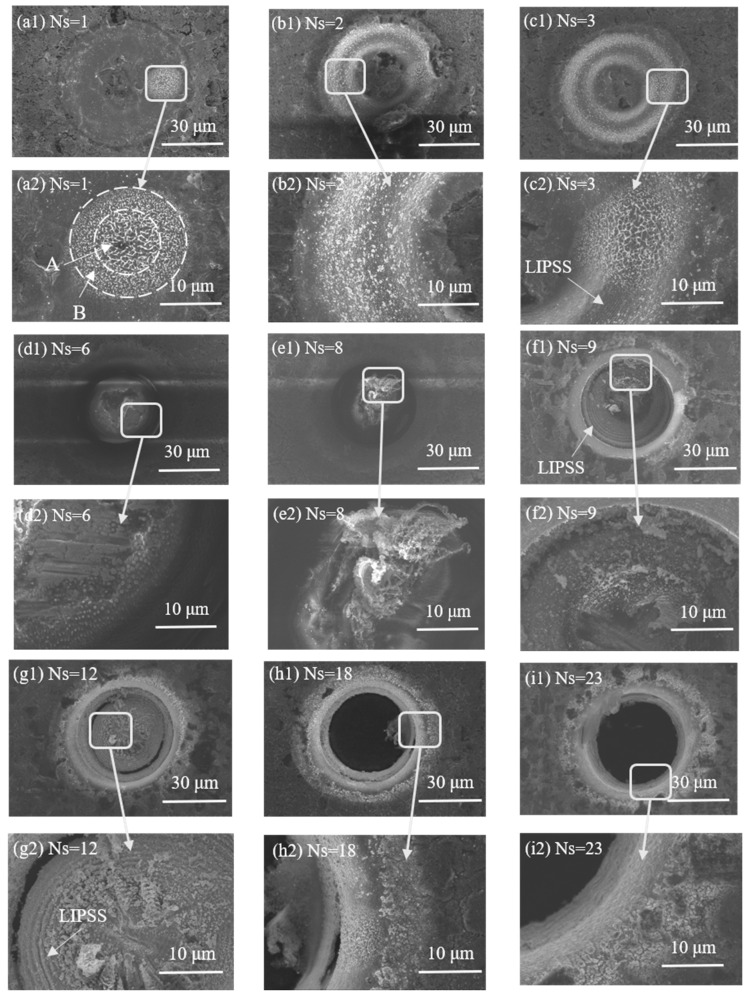
SEM analysis of micro-hole formation in FPCs processed using a femtosecond laser (E = 4 μJ, PO = 98.33%).

A small amount of ablation damage was observed on the copper as the femtosecond laser followed the circular path. Copper is primarily removed by melting. During laser processing, the laser needs to undergo a period from startup to reaching a stable output state and from shutdown to the complete disappearance of the laser, known as the on-light delay and the off-light delay, respectively. Because of these two delays, the material will absorb more laser energy at the beginning or end of laser processing. As a result, melting and solidification occur at this point, exhibiting a clear white circle, as shown in Figure 2(a1). After enlarging the circle, it can be observed that the surface ablation morphology in the laser processing area exhibits a clear gradient distribution owing to the Gaussian distribution of the laser energy. At the edge of the laser processing area, only ablation damage occurs, owing to the low energy, as shown in Figure 2(a2) A. In the middle of the processing area, the laser energy is relatively high, making part of the copper melt, as shown in Figure 2(a2) B. The surface ablation pattern in the middle of the laser processing area displays a gradient. As illustrated in Figure 2(b2), the micro-hole grooves deepen when the number of laser processing steps is tripled. Under further laser processing, the copper absorbs the energy, gradually melts, and then solidifies after cooling to form particles that adhere to the edge of the microporous grooves. A distribution of colors ranging from white to black to white is observed along the radial direction of the laser-processed path. This phenomenon is caused by the higher energy in the middle of the laser spot, which eliminates more copper, generating a Gaussian distribution of the spot during laser processing. When the number of processing times is four, as shown in Figure 2(c2), a gradient-distributed melting phenomenon still occurs at the point, and a laser-induced periodic surface structure (LIPSS) is observed on the surface copper foil.

The intermediate resin layer material becomes more visible as the number of laser processing laps increases. As shown in Figure 2(d2), at this point, the surface of the material is covered with tiny molten particles and contains both surface copper and MPI resin at the micro-hole center. As the number of laser processing steps increases, the MPI resin layer becomes abraded, producing a large amount of flocculent material that accumulates in the hole and cannot be released, as shown in Figure 2(e2).

When the femtosecond laser processes the copper at the bottom, a molten material appears in the hole. This heat-affected zone expands, outward in the radial direction owing to the continuous input of laser energy into the hole, and the contact reaction with the material increases the heat accumulation. The periodic structure remains, but the molten structure produced by the on–off delay disappears, and a significant quantity of MPI material is dispersed across the wall and bottom of the hole. This is illustrated in Figure 2(f2). The micro-holes deepen when the number of laser processing steps is 11, and a black arc appears at the bottom of the hole, as shown in Figure 2(g1), indicating that the bottom layer of copper is ready to be completely ablated at this point. As the number of processing cycles increases, the periodic structure in the form of concentric rings becomes more evident and wider, as shown in Figure 2(g2), and is always retained. A through-hole is processed using the laser when the number of processing cycles increases to 18, as shown in Figure 2(h1). However, residues that have not been eliminated remain on the hole wall. Therefore, additional processing times must be added to further process the micro-holes to improve the quality of the processing, as shown in Figure 2(i2).

#### 3.1.2. Cross-Sectional Morphology Characteristics of FPC Micro-Holes Processed Using a Femtosecond Laser

**Figure 3:**
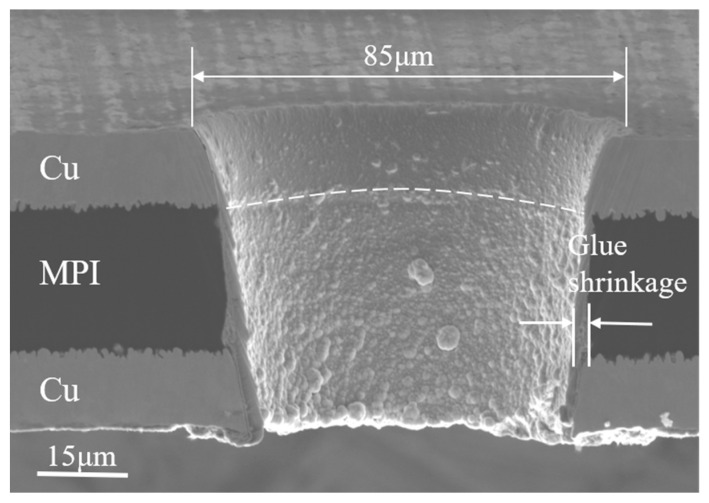
SEM analysis of the cross-sectional morphology of FPC micro-holes (E = 3.4 μJ, PO = 98.33%, Ns = 23).

Further observation and analysis of the micro-hole cross-sectional morphology were performed to clearly elucidate the principle and mechanism of femtosecond laser processing of FPCs. In Figure 3, a certain taper is observed, and the entrance diameter of the micro-holes is larger than the exit diameter; the recast layer at the entrance is less, and the pore wall of the surface copper layer is smoother, with a few tiny molten particles adhering to it. More molten particles fill the pore walls of the MPI resin and copper underneath, spreading all the way to the pore exit position. The primary reason for the molten material formation is that, while processing the bottom copper, the laser causes plasma sputtering, which diffuses the high-temperature plasma produced in the hole outward. The plasma comes into contact with the MPI resin during this process, which causes it to cool and solidify into particles connected to the resin. The figure shows that there are no visible defects, such as cracks or grooves, when the laser processes the flexible board, and MPI resin shrinkage is not noticeable.

#### 3.1.3. Material Composition Analysis of Femtosecond Laser Micro-Holes in FPC

**Figure 4:**
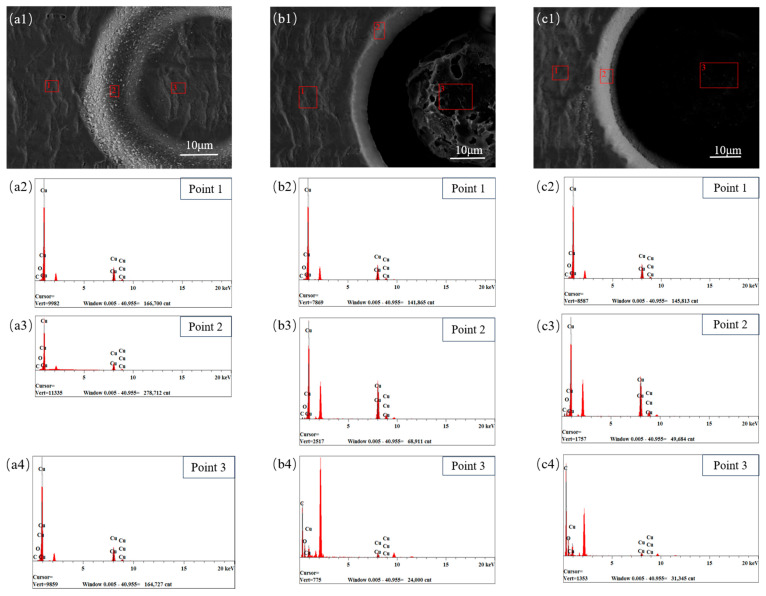
SEM and EDS analysis of femtosecond laser-machined micro-holes in FPCs (E = 3.4 μJ, PO = 98.33%, (**a**) Ns = 3; (**b**) Ns = 7; (**c**) Ns = 11).

As shown in Figure 4, energy-dispersive X-ray spectroscopy (EDS) analysis was performed on several micro-hole locations during the three stages of femtosecond laser processing of the flexible board to further determine the element changes in each area of the micro-holes. Table 3 lists the elemental distribution values. Points 1 and 2 indicate the areas that have not been treated and processed using the laser. The area where the material is removed via heat transfer and the micro-holes are not in direct contact with the laser is represented by point 3.

The radial elemental changes of the material that occur when the laser acts on the copper surface are depicted in Figure 4(a1–a4). At this point, C is not visible at points 1, 2, or 3, and no significant change is observed in the Cu content at any of the three locations. However, compared with points 1 and 3, the O content at the laser-processed point 2 is lower. This suggests that the initial copper foil surface is oxidized, and the oxide is removed during laser processing.

Figure 4(b1–b4) shows the laser processing of the MPI resin layers. According to the energy spectrum element analysis, a significant amount of MPI resin attached to the copper surface is ablated and sputtered to the surroundings during processing. At this point, the oxygen content in the hole increases compared with that in the hole wall, and a copper oxidation reaction occurs. From the outside to the inside of the hole, the C content at point 3 is much higher than that at point 2, and some copper remains at point 3.

Figure 4(c1–c4) shows the laser processing of the MPI resin layer. Large floccules and debris created by the MPI resin after laser processing, which are difficult to remove from the hole, remain in the hole when the laser processing reaches the bottom copper. Figure 4(a2) shows that the amounts of C and O at point 3 are marginally higher than those in the previous two phases; however, the Cu content is lower.

### 3.2. Characterization of Micro-Holes in FPC Femtosecond Laser Processing

**Figure 5:**
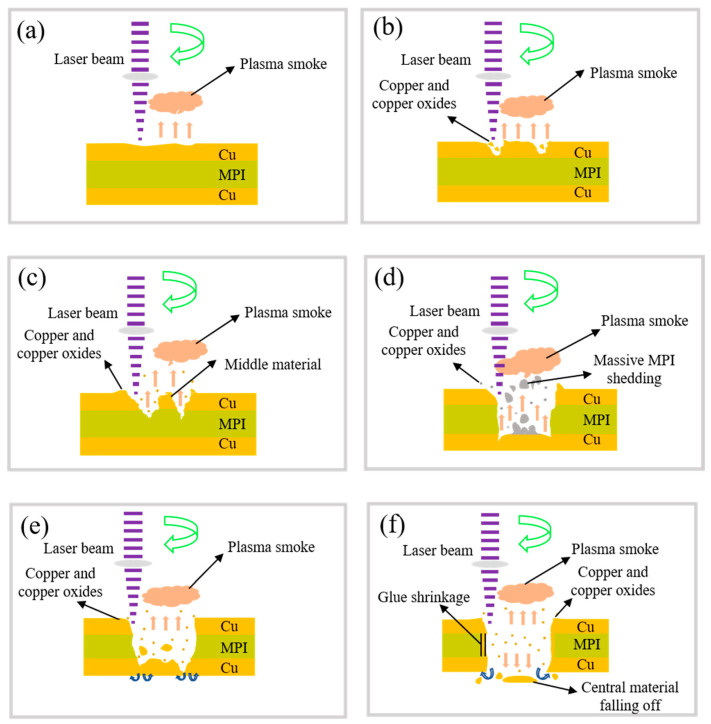
Schematic of removing micro-hole material from FPC using femtosecond laser processing. (**a**) Laser processing to the surface Cu (**b**) the surface Cu is removed (**c**) laser processing to the MPI layer (**d**) the MPI layer is removed (**e**) Laser processing to the bottom Cu (**f**) form through holes.

Three different phases may be identified in femtosecond laser processing based on an investigation of the micro-hole processing morphology of the FPC and micro-hole energy spectrum elements. When copper is processed using a femtosecond laser, as in Figure 5a, the material absorbs the laser energy and transfers it to the crystal lattice, causing the material to heat up. When the peak power density of the laser exceeds the material threshold, the material rapidly heats up internally and vaporizes, and the vaporized particles continue to absorb energy and generate ionization, forming a high-temperature, high-pressure, and high-density plasma gas. As the number of processing increases, Cur is mainly removed by melting. During laser processing, Cu reacts with oxygen in the air to form copper oxides adhering around the micro-holes, as shown in Figure 5b. At this point, there is a LIPSS structure on the copper surface.

During laser processing, copper is able to quickly transfer the absorbed laser energy to the MPI material below due to its excellent thermal conductivity. Given that the melting point of MPI is approximately 500 °C, this temperature is much lower than the melting point of copper, which is 1083 °C. Therefore, during laser processing of the copper surface layer, the MPI is likely to start melting as a result of the heat transferred from the copper. When the laser interacts with MPI, it is a dielectric, the electrons from the valence band will be excited to the conduction band, and, if the energy is high enough, they will be emitted, and plasma and ablation will be generated, as shown in Figure 5c. During laser processing, MPI also undergoes a carbonization reaction with oxygen in the air [17]. The processed MPI material is in the form of large flocculent pieces. During laser processing, copper, copper oxides, and MPI are gradually discharged and attached around the orifice, as shown in Figure 5d.

We considered that the size of the periodic surface structure correlates with the laser energy when the laser processes the bottom copper layer, unlike the surface layer of the copper. At this point, the accumulation of energy is higher, and a periodic surface structure is noticeable. Figure 5e,f shows the presence of plasma clusters near the aperture. The copper beneath the plasma cluster absorbs the laser energy and transforms into a gaseous state before being released. This process causes the temperature to decrease gradually, solidifying the copper and forming tiny particles that adhere to the wall of the MPI resin aperture. The vaporized molten copper is released downhill and sticks to the exit hole as a through-hole forms.

### 3.3. Effect of Femtosecond Laser Processing Parameters on FPC Micro-Hole Quality

#### 3.3.1. Effect of Femtosecond Laser Processing Parameters on Micro-Hole Size in FPC

(1) Effect of laser parameters on the diameters of the micro-holes.

**Figure 6:**
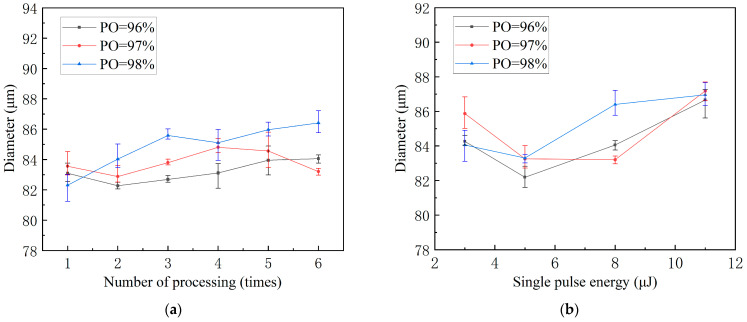
Effects of laser drilling parameters on the diameter of micro-holes. (**a**) Variations in diameter with processing times at different pulse overlap rates (E = 8 μJ); (**b**) variations in diameter with single-pulse energy at different pulse overlap rates (Ns = six times).

The diameters of the micro-holes remained at approximately 85 µm throughout laser processing, as illustrated in Figure 6a. This is because the scanning path and spot size affect the diameters of the micro-holes. The scanning route and spot size are fixed when adjusting the laser processing parameters. Some of the measured diameters are less than 85 µm because, during laser ring processing, the laser energy has a Gaussian distribution, with the highest in the middle of the spot and decreasing outward. Therefore, the energy density of the laser in the peripheral circumferential portion of the micro-holes has not yet reached the ablation threshold of the material, and the conditions for removing the material cannot be met.

The diameters of the micro-holes increased as the energy of the single pulse increased, as shown in Figure 6b. A low single-pulse energy corresponds to a low spot edge energy acting on the material with a low MRR. The energy of the spot edge acting on the material increases with an increase in the single-pulse energy, leading to a higher rate of material removal. The diameter of micro-holes surpasses the predetermined 85 µm at a single-pulse energy of 11 μJ with a PO rate of 98%. At this point, the material absorbs more energy, and a wider heat-affected zone may be generated.

(2) Effect of laser parameters on the depth of micro-holes

**Figure 7:**
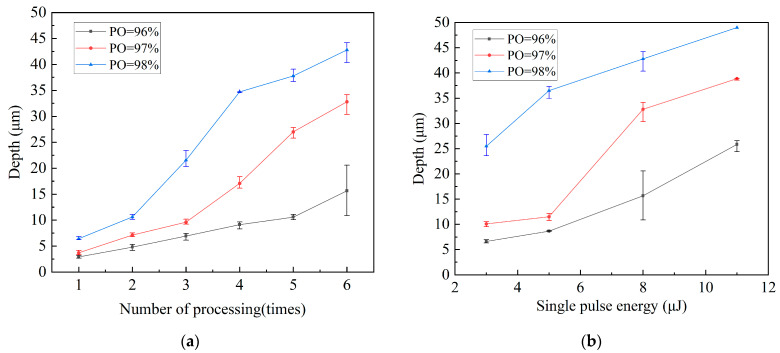
Effects of laser drilling parameters on the depth of micro-holes. (**a**) Depth variation with processing time at different pulse overlap rates (E = 8 μJ); (**b**) depth variation with single-pulse energy at different pulse overlap rates (Ns = six times).

As shown in Figure 7a, the micro-hole processing depth increases with the number of laser processing cycles. For an identical number of cycles and single-pulse intensity, more material is eliminated when the PO rate increases. According to the above result, heat transfer occurs when the laser processes the copper surface layer, warming and melting the MPI resin in advance and turning it into fine debris that sticks to the material and is difficult to remove. The processing depth is approximately 15 µm at a PO rate of 96%, and the number of processing times is six. At this point, the laser has already processed the MPI resin layer and the depth measurement is affected by uneven MPI resin debris.

The depth of the micro-holes increased with the laser single-pulse energy, as shown in Figure 7b. The micro-hole depth varies significantly when the single-pulse energy increases from 5 to 8 μJ and the PO rate reaches 97%. This is related to the material characteristics of the MPI and copper. MPI absorbs a large amount of energy during processing and removes material at a faster rate because it has a lower melting point than copper.

#### 3.3.2. Processing Parameters of Femtosecond Lasers and Effects on MRR

**Figure 8:**
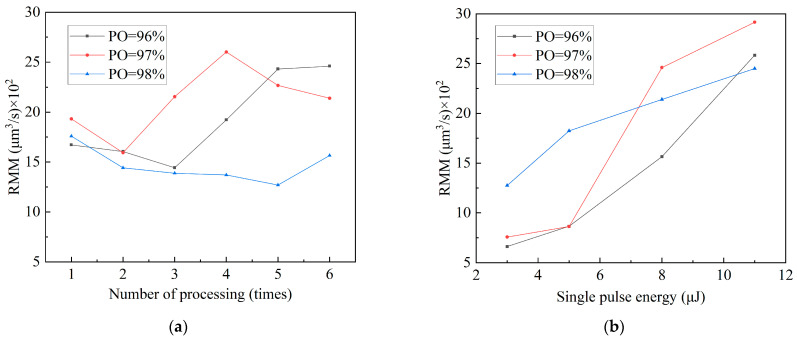
Effects of laser drilling parameters on the removal rate of micro-holes. (**a**) Material removal rate variation with processing time at different pulse overlap rates (E = 8 μJ); (**b**) material removal rate variation with single-pulse energy at different pulse overlap rates (Ns = six times).

The MRR trend under various PO rates is depicted in Figure 8a, with the rate fluctuating with the number of processing times. The figure illustrates how the MRR exhibits a tendency to first decrease, then increase, and finally decrease as the number of processing times increases for PO rates of 97% and 98%. The MRR exhibits a tendency of initially decreasing and then increasing with an increase in the number of processing sessions when the PO rate is 96%. In contrast to Figure 7, the strong correlation rate between laser processing depth and variations in the MRR is evident. The MRR steadily decreases as the number of processing cycles increases when the laser works on the copper surface of the FPC. The MRR at this point steadily increases because the melting point of the MPI resin is lower than that of copper, making it easier to remove material via laser ablation. The MRR decreases again when the bottom copper is processed using a laser.

The data shown in Figure 8b exhibit a positive correlation between the MRR and an increase in laser single-pulse energy. Under different PO rates, the MRR changes from 97% to 96% to 98%. In addition to depth variation, at a PO rate of 97%, the process removes more material, including the bottom copper, the entire MPI resin layer, and the surface copper. Surface copper and MPI resin constitute most of the processed materials when the PO rate is 96%. The melting point of MPI resin is lower than that of copper, which causes the MRR to increase gradually. The treated materials primarily consist of MPI resin and underlying copper when the PO rate is 98%, and the overall MRR increases steadily.

## 4. Conclusions

In this study, femtosecond laser technology is used to drill micro-holes in FPCs with MPI resin as a substrate, and the features of these micro-holes are examined. The main conclusions of this study are summarized below.

(1) Laser energy affects the surface morphology of copper. During the processing of the surface layer of copper, melting and solidification occur at the end of the laser path owing to the delay in turning the laser on and off, which disappears after the MPI resin layer has been processed. Throughout the entire process, the copper surface exhibited a periodic structure under the influence of laser energy, and its position remained constant.
(2) Cu reacted with airborne oxygen, thereby undergoing oxidation during laser processing. During processing, tiny oxide particles adhered to the copper surface layer. The oxides and molten MPI co-adhered to the copper surface layer during MPI processing. The MPI layer melted and ablated more quickly than the copper because of the disparate quality of the materials. The MPI formed significant amounts of flocs upon heating. The MPI generated debris that adhered to the hole wall as the processing time increased.
(3) Changes in the processing parameters did not significantly alter the diameter of the micro-holes, which was controlled by the spot size and processing path. The processing diameter of the material was affected by the outer ring of the laser spot and exhibited a Gaussian distribution, with less power transmitted at lower energies. The depth of micro-holes was directly proportional to the laser single-pulse energy, number of passes, and PO rate, which are all affected by laser processing parameters. As MPI had a lower melting point and absorbed more heat, the rate of depth change increased rapidly during processing.

## Figures and Tables

**Table 1:** Main parameters of the femtosecond laser processing system.

Project	Parameters	Units
Wavelength	343	nm
Pulse Width	500	fs
Frequency	1200	kHz
Cooling mode	thermostatic water cooling	/
Spot Diameter	20	μm
Focal length of the field lens	103	mm

**Table 2:** Experimental parameters for UV femtosecond laser processing of FPC micro-holes.

Laser Processing Parameters	Abbreviation	Experimental Parameters	Units
Single pulse energy	E	3, 5, 8, 11	μJ
Pulse overlap rate	PO	96, 97, 98	%
Number of scans	Ns	1, 2, 3, 4, 5, 6	times

**Table 3:** EDS analysis data of femtosecond laser machining of FPCs micro-holes.

Figure	Point Number	Atomic Fraction Content (%)		
		C	O	Cu
Figure 4a	1	0	1.43	98.57
	2	0	3.68	96.32
	3	0	1.24	98.76
Figure 4b	1	0	1.43	98.56
	2	1.79	0.57	97.63
	3	52.03	19.63	28.32
Figure 4c	1	0.48	2.12	97.39
	2	2.87	2.27	94.85
	3	59.99	22.63	17.37

## Data Availability

The original contributions presented in the study are included in the article, further inquiries can be directed to the corresponding author.
